# Simultaneous Lymphatic Superficial Circumflex Iliac Artery Perforator Flap Transfer from the Zone 4 Region in Autologous Breast Reconstruction Using the Deep Inferior Epigastric Artery Perforator Flap: A Proof-of-Concept Study

**DOI:** 10.3390/jcm11030534

**Published:** 2022-01-21

**Authors:** Hidehiko Yoshimatsu, Ryo Karakawa, Yuma Fuse, Tomoyuki Yano

**Affiliations:** Cancer Institute Hospital of the Japanese Foundation for Cancer Research, Department of Plastic and Reconstructive Surgery, 3-8-31 Ariake, Koto-ku, Tokyo 135-8550, Japan; ryo.kyara@gmail.com (R.K.); yuyuma.fuse@gmail.com (Y.F.); yanoaprs@icloud.com (T.Y.)

**Keywords:** lymphedema, deep inferior epigastric artery perforator flap, breast reconstruction, superficial circumflex iliac artery perforator flap

## Abstract

The incidence of upper extremity lymphedema after breast cancer treatment is reported to be 14% after axillary lymph node dissection (ALND) and 33% after ALND and regional lymph node dissection. The present report describes a novel method in which the afferent lymphatic vessels are harvested with their lymph nodes from the Zone 4 region as a separate flap, the superficial circumflex iliac artery perforator (SCIP) flap, in the setting of autologous breast reconstruction using the deep inferior epigastric artery perforator (DIEP) flap. From September 2017 to September 2020, seven female patients with an average age of 46.9 years (range: 39 to 54 years) underwent autologous breast reconstruction using the DIEP flap and the lymphatic SCIP flap procured separately from the Zone 4 region. All patients had undergone ALND, four patients had undergone radiation therapy, and three patients had established lymphedema at the time of reconstruction. All lymphatic SCIP flaps survived completely. Lymphedema did not occur in any of the four patients to whom the lymphatic flap was transferred for a preventive purpose (average follow-up: 37.5 months). In three patients with established lymphedema at the time of reconstruction, the average rate of estimated volume decrease at the last follow-up (average: 29.0 months) was 12.6%. A lymphatic SCIP flap procured from the Zone 4 region in DIEP flap breast reconstruction can contribute to improvement or prevention of lymphedema with no additional donor site.

## 1. Introduction

The incidence of upper extremity lymphedema after breast cancer treatment is reported to be 14% after axillary lymph node dissection (ALND) and 33% after ALND and regional lymph node dissection [1]. Treatment of lymphedema comprises complete decongestive therapy, lymphovenous anastomosis (LVA), vascularized lymph node transfer (VLNT), and debulking procedures, but the optimum indications are yet to be defined [2,3,4].

The deep inferior epigastric artery perforator (DIEP) flap is the most widely used flap for autologous breast reconstruction. DIEP flap transfer including the lymph nodes in the lower abdomen has been reported to alleviate lymphedematous limbs [5,6,7]. Because the inguinal lymph nodes are often nourished by the superficial circumflex iliac artery (SCIA) and the superficial circumflex iliac vein (SCIV), additional anastomoses become necessary to achieve satisfactory efficacy of the lymph node transfer [8,9].

In this report, we introduce a novel method in which the afferent lymphatic vessels are harvested with their lymph nodes from the Zone 4 region as a separate flap, the superficial circumflex iliac artery perforator (SCIP) flap, in the setting of autologous breast reconstruction using the DIEP flap. This lymphatic SCIP flap is then transplanted to the axillary region for treatment or prevention of lymphedema.

## 2. Materials and Methods

Institutional Review Board (IRB) approval was obtained (2021-GB-052). From September 2017 to September 2020, 7 female patients with an average age of 46.9 years (range: 39 to 54 years) underwent autologous breast reconstruction using the DIEP flap and the lymphatic SCIP flap procured separately from the Zone 4 region. All patients had undergone ALND, 4 patients had undergone radiation therapy, and 3 patients had established lymphedema at the time of reconstruction. Patients’ demographic data are given in Table 1. All patients who underwent ALND at our institution were followed regularly by certified lymphedema therapists. At each visit, circumferences were measured at the following sites: the axilla, 5 cm cephalad to the elbow, the elbow, 5 cm caudal to the elbow, the wrist, and the dorsum. A diagnosis of lymphedema was made when a patient reported symptoms of lymphedema and demonstrated a 10% increase in relative volume change. The relative volume change was calculated by dividing the pre- and post-operative difference by the preoperative value. Estimated limb volume was calculated using the following frustum formula: V = (d)(A2 + Aa + a2)/12(π), where “A” is the circumference measurement of the distal section, “a” is the circumference measurement of the proximal section, and “d” is the distance between the distal and the proximal section [10].

### Surgical Technique

The flap is elevated in a prone position. Preoperatively, the course of the superficial branch of the SCIA and the location of the DIEP are marked using an ultrasound device [11,12]. The DIEP flap is elevated in an ordinary fashion. Reverse mapping using indocyanine green (ICG) lymphography is performed as described in a previous report [13]. The afferent lymphatic vessels and their draining lymph nodes are then identified using ICG lymphography, injecting ICG intradermally into the region [14]. The superficial branch of the SCIA is found and dissected through the caudal edge of the flap, as described in our previous report [15]. After confirmation of perfusion to the lymph nodes, a SCIP flap is designed, incorporating the corresponding lymphatic vessels. The thoracodorsal artery and its accompanying vein, or the serratus branch and its accompanying vein, are prepared as the recipient vessels. The lymphatic SCIP flap is transplanted before the DIEP flap transfer because the elevation of the lymphatic SCIP flap takes less time than that of the DIEP flap. After microsurgical anastomoses, the skin paddle is de-epithelialized, and the distal portion of the flap is sutured to the proximal upper arm, considering the axiality of the lymphatic vessels in the flap. The perfusion of the embedded lymphatic flap is monitored postoperatively using ultrasonography devices.

## 3. Results

The characteristics of the patients and the reconstruction summary are shown in Table 1. The mean body mass index was 23.7 (range: 20.3 to 28.2). The average size of the flap was 12.4 × 5.3 cm (range: 9 × 4 cm to 20 × 8 cm). The thoracodorsal artery was used as the recipient artery in four cases, the serratus branch of the thoracodorsal artery in three cases, and a branch of the deep inferior epigastric artery in one case. Average follow-up length was 33.9 months (range: 13 to 48 months). Lymphedema did not occur in any of the four patients to whom the lymphatic flap was transferred for a preventive purpose (average follow-up: 37.5 months). In three patients with established lymphedema at the time of reconstruction, the average rate of estimated volume decrease at the last follow-up (average: 29.0 months) was 12.6%. These findings are summarized in Table 1.

### Case

**Figure 1:**
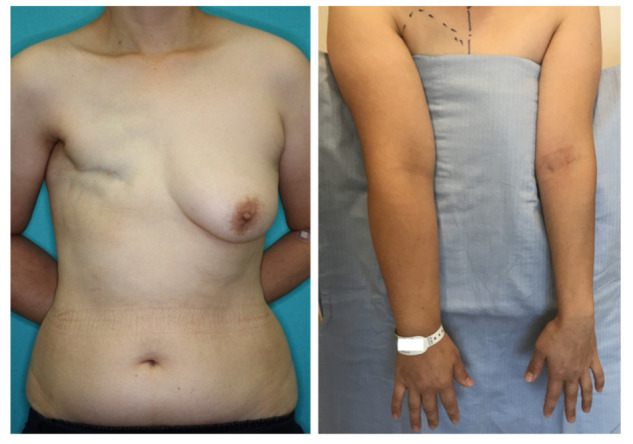
A 46-year-old woman was diagnosed with an International Society of Lymphology stage 2B lymphedema of the right upper extremity after undergoing mastectomy, ALND, and preoperative radiation therapy. ALND, axillary lymph node dissection.

**Figure 2:**
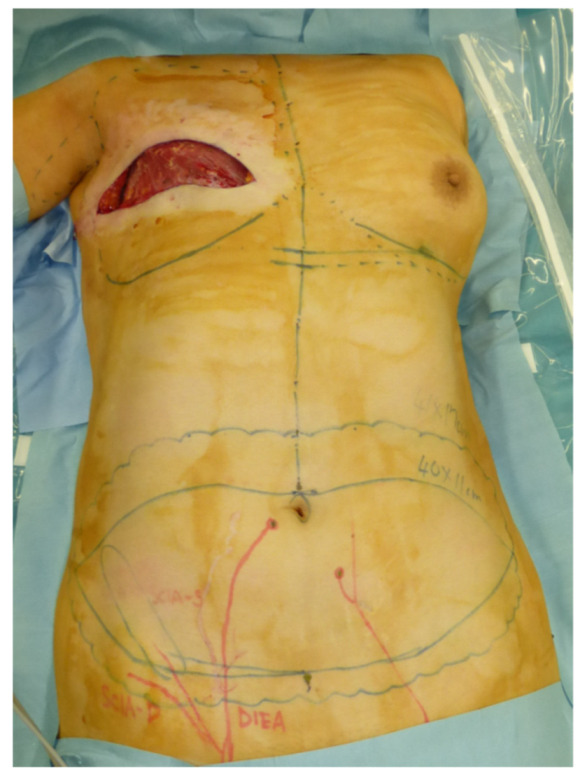
A DIEP flap was designed in the left hemiabdomen, and a 14 × 5 cm lymphatic SCIP flap was designed in the Zone 4 region. DIEP, deep inferior epigastric perforator; SCIP, superficial circumflex iliac artery perforator.

**Figure 3:**
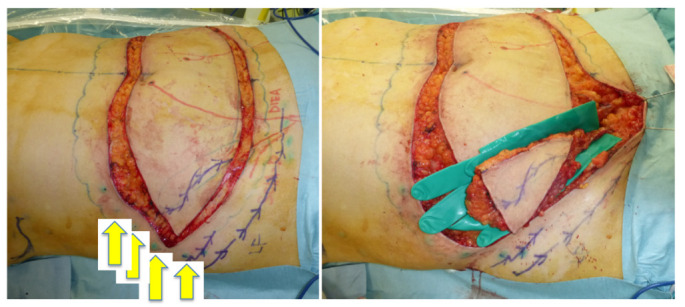
Left: ICG was injected into multiple sites (yellow arrows) in the lateral hemiabdomen. The lymph nodes and the corresponding lymphatic vessels (blue lines) were visualized and marked using ICG lymphography. Right: the lymphatic SCIP flap was elevated, incorporating the lymph nodes and their corresponding lymphatic vessels. ICG, indocyanine green; SCIP, superficial circumflex iliac artery perforator.

**Figure 4:**
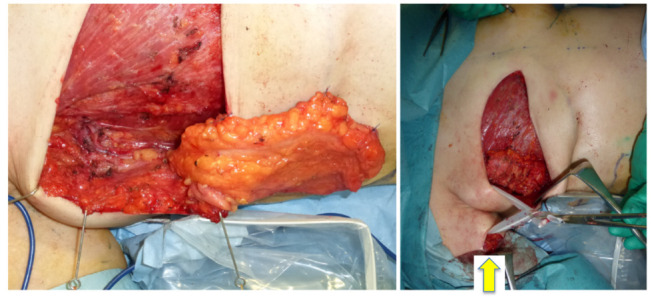
Left: the SCIA and the SCIV were anastomosed to the thoracodorsal artery and its accompanying vein, respectively. Right: the flap was de-epithelialized and the distal end of the flap was sutured to the axillary region (yellow arrow), considering the axiality of the lymphatic pathway. SCIA, superficial circumflex iliac artery; SCIV, superficial circumflex iliac vein.

**Figure 5:**
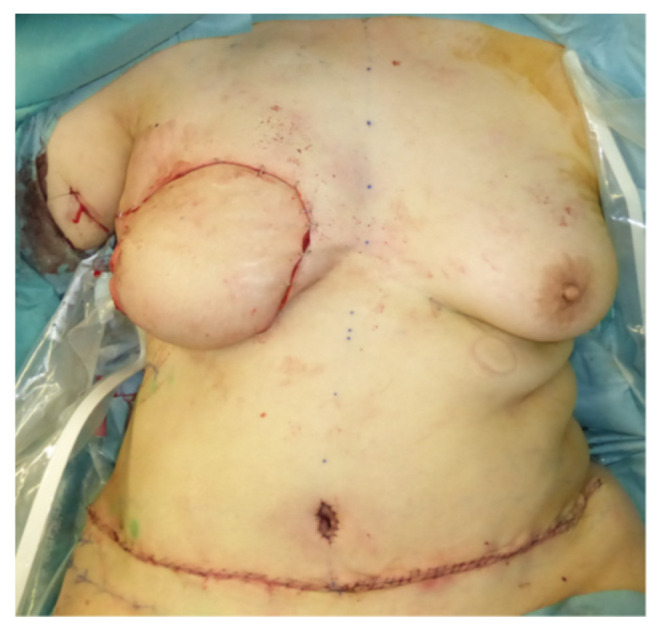
The breast was reconstructed using the DIEP flap. DIEP, deep inferior epigastric perforator.

**Figure 6:**
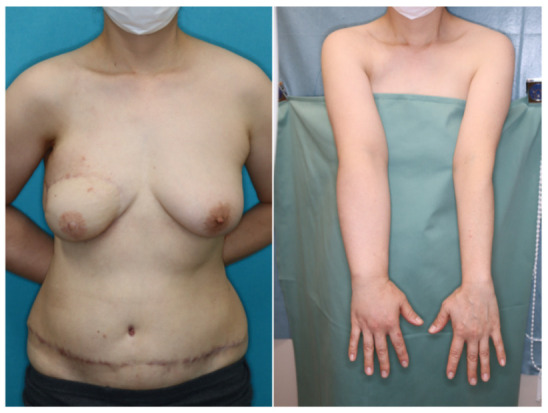
The estimated volume had decreased by 12.6% at 48 months postoperatively.

A 46-year-old woman was diagnosed with an International Society of Lymphology (ISL) stage 2B lymphedema of the right upper extremity after undergoing mastectomy, ALND, and preoperative radiation therapy (Figure 1). A DIEP flap was designed in the left hemiabdomen, and a 14 × 5 cm lymphatic SCIP flap was designed in the Zone 4 region (Figure 2). ICG was injected into multiple sites (designated by the yellow arrows) in the lateral hemiabdomen. The lymph nodes and the corresponding lymphatic vessels were visualized and marked using ICG lymphography (Figure 3, left). The lymphatic SCIP flap was elevated, incorporating the lymph nodes and their corresponding lymphatic vessels (Figure 3, right). The SCIA and the SCIV were anastomosed to the thoracodorsal artery and its accompanying vein, respectively. (Figure 4, left). The flap was de-epithelialized, and the distal end of the flap was sutured to the axillary region, considering the axiality of the lymphatic pathway (Figure 4, right). The breast was reconstructed using the DIEP flap (Figure 5). The estimated volume had decreased by 12.6% at 48 months postoperatively. (Figure 6).

## 4. Discussion

The treatment and management of breast-cancer-related lymphedema has been challenging. Recently in many institutions, LVA has been indicated for early-stage lymphedema where functional lymphatic vessels remain, and VLNT for advanced-stage lymphedema in which most lymphatic vessels have lost their functionality [2,3,4]. Among many controversies regarding lymphedema treatment, a consensus has been reached that “the earlier, the better”. Thus, the importance of immediate lymphatic reconstruction has recently been emphasized [16,17].

The DIEP flap transfer with inguinal lymph nodes has been widely advocated because it can kill two birds with one “flap” [5,6,7,8,9]. However, the inguinal lymph nodes are sometimes perfused by the superficial arteries, the superior epigastric artery or the SCIA, in which cases, additional anastomoses become essential for the lymph nodes to retain their function. Another disadvantage of the DIEP flap transfer with inguinal lymph nodes is that it is almost impossible to reconstruct the lymphatic pathway, considering the axiality of the flap, which has been recently proposed as an essential aspect for lymphedema treatment [18].

The novelty of this method lies in the fact that lymphatic flow is also reconstructed in the proposed approach. This is because approximately 10 cm of the afferent lymphatic vessels are being transferred with their lymph nodes, which creates a new pathway from the proximal arm. The lymphatic flap has been previously described in one report, and this is the first paper to describe the use of the lymphatic flap simultaneously in autologous breast reconstruction using the DIEP flap [14].

There are many advantages of this lymphatic SCIP flap transplant performed simultaneously with the DIEP flap breast reconstruction. Reconstruction of the lymphatic pathway via the lymphatic vessels and their corresponding lymph nodes implies that this method could be adopted for immediate lymphatic reconstruction, along with immediate breast reconstruction using the DIEP flap. Although we did not have immediate reconstruction in our case series, lymphedema did not occur in any of the four patients who underwent preventive lymphatic SCIP flap transplant performed simultaneously with the DIEP flap breast reconstruction. This number of patients is too small to deduce any conclusion, but improvements in the lymphedematous patients could suggest its preventive potential when applied to immediate lymphatic reconstruction. In addition to creating a new lymphatic pathway, the SCIP flap also prevents scar tissue formation in the axillary region, which can cause edema from both lymphatic and venous congestion. In patients with established lymphedema, it is crucial to remove the scar tissue and place a well-perfused flap in the axillary region [4]. From a cosmetic perspective, this additional flap transplant fills the dead space created after ALND, which is not possible with the DIEP flap transfer with inguinal lymph nodes. Furthermore, a new donor site is not necessary in this lymphatic flap transplant. In many cases, the Zone 4 region of a DIEP flap will otherwise be discarded. However, the lymphatic vessels and lymph nodes are procured from the groin region, and thus additional precautions should be taken to prevent donor-site complications. Seroma is the most common complication, and lower extremity lymphedema is a rare but detrimental complication after procurement of the lymph nodes in the groin region [5,6,19,20]. Neither complication was seen in this case series, and we believe reverse mapping using ICG lymphography plays a crucial role in the prevention of these complications [13].

This study is limited by the fact that the paucity of the number of cases precludes drawing any definite conclusions and that the study did not include a control group. Further comparative study with a large number of patients is required. The short follow-up period is also a shortcoming because symptoms of lymphedema usually manifest three to four years after ALND. Another limitation of this method is that this simultaneous lymphatic flap tranfser cannot be performed in cases with bilateral breast reconstruction, nor can this method be applied in cases where a large DIEP flap including the Zone 4 region is required for breast reconstruction.

## 5. Conclusions

A lymphatic SCIP flap procured from the Zone 4 region in DIEP flap breast reconstruction can contribute to the improvement or prevention of lymphedema with no additional donor site.

## Figures and Tables

**Table 1:** Patient characteristics and reconstruction summary.

Patient	Age	BMI	Rx	ALND	ISL Stage	Flap Size (cm)	Recipient Artery	Volume Decrease Rate at the Last Follow-Up (%)	Follow-Up (months)	Lymphedema Occurrence
1	46	23.2	+	+	2B	14 × 5	Thoracodorsal artery	16.3	48	N/A
2	44	20.3	-	+	0	16 × 5	Serratus branch of TDA	N/A	48	-
3	39	28.2	+	+	0	9 × 4	Serratus branch of TDA	N/A	45	-
4	54	21.4	-	+	0	12 × 4	Branch of DIEA	N/A	33	-
5	45	26.8	-	+	2B	14 × 5	Thoracodorsal artery	8.9	26	N/A
6	49	23.7	+	+	0	20 × 8	Thoracodorsal artery	N/A	24	-
7	51	22.2	+	+	2A	16 × 6	Serratus branch of TDA	18.5	13	N/A
Average	46.9	23.7				12.4 × 5.3		12.6	33.9	

BMI, body mass index; Rx, radiation therapy; ALND, axillary lymph node dissection; TDA, thoracodorsal artery; DIEA, deep inferior epigastric artery; N/A, not applicable.

## Data Availability

The data that support the report are available from the corresponding author, H.Y., upon reasonable request.
